# Ending Epidemics: A History of Escape from Contagion

**DOI:** 10.3201/eid3102.241462

**Published:** 2025-02

**Authors:** Clyde Partin

**Affiliations:** Emory University Orthopaedics & Spine Hospital, Tucker, Georgia, USA

**Keywords:** epidemics, book review

In 310 pages, the author tells the “story of humanity’s long and, until now, extraordinarily successful struggle against infectious diseases” and weaves together 3 centuries of progress in managing infectious disease (Figure). The 31 chapters are presented chronologically, each typically recounting 1 or 2 diseases, key scientists or physicians involved in conquering the contagion, and their methods. Chapter 9 reminds us that the foundation of public health is not necessarily the image of the physician-scientist triumphantly standing with each pathogen discovery. Real progress had more to do with confronting mundane forces of hygiene, primarily disposal of human waste and the advent of the sanitation revolution.

**Figure:**
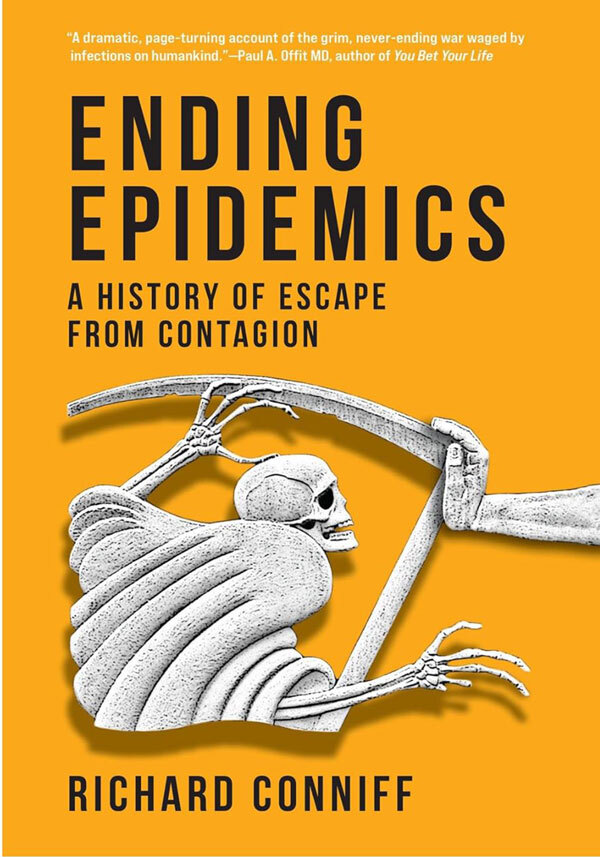
**Figure.** Ending Epidemics: A History of Escape from Contagion

The author ends the book with the last known case of smallpox, in Janet Parker, who died in 1978. Inevitably, he confronts COVID-19 in the epilogue, juxtaposing pessimistic caution, “the reality is that victory over infectious diseases was never a done deal,” with an idealistic goal, “the job now is to protect not just the human species but the world itself. It will take ‘delusional optimism’ on a global scale.”

The Yale-educated author is a decorated journalist, with a record of publishing science articles about human and animal behavior and nature. The writing style is lively, convincing, and spiced with literary references. Fascinating trivia is plentiful. For example, Russian zoologist Metchnikoff was so enamored of his time at Institut Pasteur that he asked to be cremated with the research animals. The personal life of these researchers is also explored.

Chapters 12 and 13 are particularly well done, introducing nuanced background information. Italian physician/microscopist Filippo Pacini did much to elucidate the pathologic nature and cause of cholera. He named the organism *Vibrio cholerae *in an 1854 article, as John Snow was gaining notoriety. Reverend Henry Whitehead was a keen observer and “unexpectedly proved to be a better epidemiological detective,” having recognized the ground zero cholera case in a 5-month-old girl. Whitehead, a man of the cloth more than science, was an underappreciated sleuth whose astute observations were essential in understanding a cholera outbreak. 

Several underheralded figures introduced by the author include Reverend Cotton Mather and Isabel Morgan. Mather, a controversial clerical figure in Boston, was interested in medicine and science. When smallpox landed in Boston, Mather was aware of the practice of variolation, which he learned from his servant, Onesimus, who was a native of Ghana. Identifying Bostonians born in Africa with variolation scars helped Mather overcome initial resistance from the Boston medical community to the technique. Morgan, a brilliant researcher, left the Rockefeller Institute because advancement for a woman was not likely there. In a polio group at Johns Hopkins University, she pioneered an inactivated poliovirus, refuting the dogma that only a live vaccine would work.

Topics such as contagionism, germ theory, antivivisection, and spontaneous generation are expertly woven into the narrative. References to misogynist statements will make the reader pause.

This author condensed >3 millennia of human misery related to infections, plagues, and poor sanitation, followed by a cogent narrative chronicling the ingenuity of physician-scientists and others who solved those problems. More depth and greater insight are provided than typical with the historical scenarios the author has presented.

